# Of the Epidemic Catarrh of the Year 1788

**Published:** 1788

**Authors:** Samuel Foart Simmons


### LONDON MEDICAL JOURNAL.

### I. *Of the epidemic Catarrh of the Year* 1788. *By* Samuel Foart Simmons, *M. D. F. R. S.*

IN ſome of the earlieſt inſtances I had occaſion to ſee of this epidemic in London, the patients dated their complaints from the latter end of June; but the diſeaſe could hardly be ſaid to occur with much frequency before the ſecond week of July; from which period, till about the fourth week of that month, ſeemed to be the ſpace of time in which it was moſt prevalent. In the beginning of Auguſt it was evidently become much leſs frequent; but it ſtill continued to appear during that and the two ſucceeding months, and two inſtances of it occurred to me even ſo lately as the middle of November*.

The

* From a pretty accurate regiſter of two hundred and thirty-five caſes in which I had an opportunity of obſerving this diſeaſe, (one hundred and ſixty of which occurred at the

Weſtminſter

The weather, for ſome weeks preceding the appearance of the epidemic in this country, had been remarkable only for its dryneſs; and to this ſucceeded frequent rains from the latter end of June till the middle of July. Some degree of prediſpoſition to the diſeaſe might perhaps be occaſioned by this change in the ſtate of the atmoſphere; but it ſeems now to be pretty generally acknowledged that the origin of the epidemics of this ſort, which have at different periods ſpread over conſiderable parts of the world, and of courſe through different climates, is not to be ſought for in any of the ſenſible qualities of the air: and in the late epidemic, as in former diſeaſes of the ſame kind, many facts oc-

Weſtminſter General Diſpenſary) it appears that of this number,

| | | | | | | | |
|---|---|---|---|---|---|---|---|
| From | June | 23 | to | July | 7, both days included, | 15 | Perſons were attacked with it. |
| —— | July | 8 | — | —— | 21, | 79 | |
| —— | —— | 22 | — | Aug. | 4, | 32 | |
| —— | Aug. | 5 | — | —— | 18, | 23 | |
| —— | —— | 19 | — | Sept. | 1, | 12 | |
| —— | Sept. | 2 | — | —— | 15, | 21 | |
| —— | —— | 16 | — | —— | 29, | 18 | |
| —— | —— | 30 | — | Oct. | 13, | 22 | |
| —— | Oct. | 14 | — | —— | 27, | 7 | |
| —— | —— | 28 | — | Nov. | 14, | 6 | |

curred curred tending to corroborate the opinion of its being propagated by contagion.

Like the influenza of the year 1782, it prevailed in the northern parts of Europe ſeveral weeks before it was felt in this country; and the following account is given in the *Gazette Salutaire* of May 29, 1788, of its effects at Warſaw and Cherſon, though without aſcertaining the date of its appearance in either of thoſe places.

" Letters from Warſaw mention, that the

" ſame catarrhal affection which, in 1782, pre-

" vailed throughout Europe, under the name

" of Influenza, has again made its appearance

" in that capital. The King of Poland and at

" leaſt two thirds of the inhabitants have been

" or are at preſent affected with it; and though

" few perſons have died, the greater number are

" confined by it to their beds. Theſe letters

" add, that a diſeaſe of this ſort, occaſion-

" ed principally by variations in the tempera-

" ture of the air from heat to cold, and com-

" bined with a good deal of humidity, could

" not fail to ſpread throughout Poland and even

" to the frontiers; ſo that the armies had not

" been exempt from it. It prevails particu-

" larly at Cherſon, where the deaths of ſome

" perſons

" persons in consequence of it at first gave rise " to a supposition that it was an inflammatory " contagion, or even the plague *."

According to this account the disease spread from Warsaw to the armies on the frontiers of Poland, and from thence to Cherson; but when we consider that the complaint is spoken of as being actually prevailing at Warsaw at the time the letters were written, and compare this with what is said of the mortality it had already occasioned at Cherson, it seems more reasonable to suppose that the epidemic had appeared first in the latter place, the proximity

* " Des lettres de Warsovie portent que la meme maladie " catarrhale qui a regné en 1782 dans toute l'Europe sous le " nom d'*Influenza* recommence a y faire des ravages. Le " Roi de Pologne & les deux tiers au moins des habitans de " cette capitale en ont été ou en sont attaqués; & quoique " peu de gens en meurent, la plupart sont obliger de garder " le lit. Ces lettres ajoutent qu'une pareille maladie, causée " principalement par la temperature d'un air variable du " chaud au froid & melé de beaucoup d'humidité, n'a pu " manquer de s'etendre par toute la Pologne & jusqu'aux " frontieres; de sorte que les armées n'en sont pas restées ex- " emptes. Elle regne surtout a Cherson, on quelques acci- " dens de personnes qui en sont mortes, ont d'abord fait " croire que c'etoit une contagion inflammatoire, ou meme la " peste."

of of which to Aſia renders it probable that it had prevailed in that quarter of the globe previouſly to its appearance in Europe, as was the caſe with the epidemic of the year 1782, and probably with other epidemics of the ſame kind.

From a later number of the ſame work we learn that the epidemic began to be felt, about the middle of April, at Vienna, where, before the 20th of that month, more than twenty thouſand perſons were ſuppoſed to be affected with it; and that it went on increaſing till about the 25th; after which time it began to diminiſh in frequency. In this account alſo, which is ſaid to be copied from the Literary Gazette of Ratiſbon, mention is made of its having already been very general in the northern parts of Europe, particularly in Ruſſia and Poland *.

It

* "VIENNE. La fievre catarrhale qui, comme on ſcait "par les feuilles publiques, a attaqué tant d'hommes dans les "pays du nord, principalement en Ruſſie & en Pologne, & "laquelle à l'inſtar de la maladie analogue épidemique en "1782 voyage de pays en pays, s'eſt actuellement repandue "comme un nuage du nord eſt ſur notre contrée. Elle a com-"mencé vers la mi d'Avril d'attaquer pluſieurs perſonnes; le "20, il y eut deja dans cette ſeule ville plus de 20,000 indi-"vidus qui en etoient affectés, & le nombre des malades al-"loit toujours en augmentant juſqu'au 25. Depuis ce jour

It did not reach Munich till the month of June *.

At Paris it began to be perceived towards the middle of Auguſt †, and had not entirely ſubſided on the 24th of October, as I learn from a letter of that date with which M. Vicq D'Azyr has favoured me.

At Geneva, as I find from a letter which Dr. Blagden has had the goodneſs to communicate to me from Dr. Odier of that city, it appeared

" ſes ravages ont paru diminuer."—*Gazette Salutaire*, 6 Novembre, 1788.

* " MUNICH, *du Mois de Juillet*. Il y'a pres d'un mois " que l'influenza, comme on l'appelle, s'eſt manifeſtée ici " avec ſes differens ſymptomes." — *Gazette Salutaire*, 6 Novembre, 1788.

† The account given of it in the *Journal de Medecine* is as follows :—" Le ciel, fréquemment chargé de gros nuages, " a donné beaucoup de pluie par averſes, du douze au trente- " un (d'Août), & leur paſſage s'eſt fait vivement ſentir ſur " les corps animés, quoique les hygromètres & les thermo- " mètres y fuſſent peu ſenſibles. Cette conſtitution a multi- " plié les affections ſéreuſes, & entretenu les bilieuſes & les " ſéro-bilieuſes. Les premières, deſquelles peu de perſonnes " ont été exemptes, dérivant de la tranſpiration dérangée, ont " donné des rhumes, des fluxions, des courbatures, & des " devoiemens ſimples ; celles-ci ſe ſont jugées aſſez prompte- " ment, en procurant une tranſpiration ſoutenue par les dé- " layans légèrement diaphorétiques." — *Journ. de Medecine*, Octobre 1788, page 101.

about about the 10th of October; and this is the lateſt intelligence I have received relative to its progreſs on the Continent.

This diſeaſe was obſerved in ſome parts of Kent, and in particular on board a guardſhip at Chatham, in the ſecond week of July; but at Kilburn, a village only two miles diſtant from London, on the Edgware road, no inſtance of it appeared to have occurred before the 19th of that month.

It began in Dover Caſtle on the 15th or 16th of July, and went through the garriſon in a ſhort time; but did not appear in the town before the 21ſt of July. For this fact I am indebted to Dr. Blagden and the Rev. Mr. Lyon.

Of the date of its appearance at York I have not been informed; but I know, from very reſpectable authority, that it had not been felt there on the 5th of Auguſt *: and yet at that very time it was preſent at Harrowgate, in the

* Dr. Hunter, a very experienced phyſician at York, in a letter to me, dated Auguſt 5, ſays, "We have not had the "ſlighteſt appearance of a catarrh in our city or neighbour- "hood during this year. I have indeed one patient who la- "bours under a mucous expectoration, which ſhe ſays ſhe "caught in London about ſix weeks ago, but the diſeaſe is "almoſt worn away."

ſame county. A gentleman, who quitted the latter place on the 7th of Auguſt, and who had been ſlightly affected with the diſeaſe, aſſured me that it had prevailed there ſeveral days before his departure.

It did not appear at Mancheſter before the latter end of July *; nor in Cornwall † till the middle

* See the account given of this diſeaſe, in the next article, by Dr. Bew.

† For the date of its appearance in Cornwall I am indebted to Dr. William May, Phyſician at Truro, who has favoured me with the following account of the diſeaſe as it occurred in that part of the kingdom :

" The firſt appearance of the late epidemical catarrh, in " this part of the kingdom, was towards the middle of Au- " guſt, and it became very general in the ſpace of two or " three weeks. The ſum of my obſervation with reſpect to " this diſeaſe is, that it was in general conſiderably milder " than the epidemic of 1782 ; that it differed from the com- " mon catarrh only by the intenſeneſs of the ſymptoms of " coryzo and raucedo which accompanied it ; and that the py- " rexia, which in moſt caſes was very ſlight, ſeldom conti- " nued longer than the fourth or fifth day, being removed, " in the greater number of inſtances, by a free determination " to the ſkin. There was, indeed, a pretty general ſymptom " which I do not recollect to have taken notice of in the pre- " ceding influenza, namely, a rheumatic affection, chiefly " confined to the ſuperior part of the body, and which, in " ſome caſes, ran out into diſagreeable lengths, putting on the

" appearance middle of Auguſt; about which period alſo, according to an account inſerted in different newſpapers,

" appearance of true chronic rheumatiſm. There was alſo " one patient of mine who had a typhus ſuperinduced upon " the influenza, (as it clearly appeared to be in the firſt in- " ſtance) and I was not a little ſurpriſed to find that the in- " flammation affecting the mucous membrane of the fauces " produced an exulceration of the tonſils, and that the diſeaſe " took on the nature of a cynanche maligna, requiring bark " and cordials to be liberally adminiſtered. What aſtoniſhed " me, in this caſe, was the ſpeedy tranſition from a ſtate of " phlogiſtic diatheſis to ſo oppoſite a condition of the ſyſtem " as that which favoured the acceſs of a typhous fever.

" With reſpect to the ſtate of the weather, I lament that " I had not time to keep any account of it; but, as far as my " recollection goes, there was little of variation from a mild, " temperate condition of the atmoſphere. There is one cir- " cumſtance more which I think worthy of remarking, and " which probably may throw ſome light on the remote cauſes " of the diſeaſe. During the progreſs of the influenza, a " complaint, which was evidently an inflammatory affection " of the mucous membrane of the fauces, &c., was frequently " obſerved among horſes, and other cattle, and was generally " as violent among them as it was mild among their rational " neighbours. In many inſtances, after an illneſs not longer " than four or ſix days, horſes were found to die, and where " this happened, it was for the moſt part attended with ſymp- " toms of violent pneumonic inflammation. Indeed I have " been informed by an ingenious ſurgeon of my acquaintance, " who does not find himſelf degraded by attending to the

" complaints newfpapers, it prevailed very generally at Aberdeen. At Montrofe, as I am informed by Mr. T. Chriftie, it was firft perceived towards the latter end of Auguft, at which time it was very mild, and few perfons had it; but about the fecond week of October it prevailed with greater violence, and was much more general than before.

In no inftance that came within my knowledge did it attack a whole family at once, but in general they became affected with it fucceffively. In one family of thirty-nine perfons, for inftance, feventeen of whom had it, the firft who experienced it was attacked on the 3d of July, and the laft not before the 1ft of September; and in St. Luke's Hofpital inftances of it continued to occur from the 16th of July till the 10th of November*.

A lady

" complaints of this ufeful fpecies, that he directed venæfection in moft cafes, and had always obferved a very confiderable inflammatory fize on the furface of the blood, and " that upon the repetition of this evacuation relief was always obtained."

* The number of perfons in the hofpital during the abovementioned period was about one hundred and ninety; but among thefe the difeafe was fo far from being general, that I

faw

A lady who came from Suffolk on a viſit to a family in London, on the 23d of July, found ſeveral perſons of the family labouring under the diſeaſe. She herſelf was ſeized with it on the 30th, and on the 1ſt of Auguſt ſhe returned

ſaw only twenty-five inſtances in which it was diſtinctly marked. It is probable, however, that, beſides theſe, there were many of the patients who had it in ſo ſlight a degree as not to excite attention, or who were incapable of deſcribing their complaints. In the above-mentioned twenty-five caſes the dates of the commencement of the diſeaſe were as follows, viz.

| | | | | |
|---|---|---|---|---|
| July – – | 16 | in | 4 | caſes. |
| | 18 | — | 2 | |
| | 28 | — | 1 | |
| Auguſt – | 8 | — | 1 | |
| | 11 | — | 1 | |
| | 20 | — | 1 | |
| September | 3 | — | 1 | |
| | 6 | — | 1 | |
| | 18 | — | 1 | |
| | 19 | — | 1 | |
| | 26 | — | 1 | |
| | 29 | — | 2 | |
| October – | 5 | — | 1 | |
| | 6 | — | 2 | |
| | 27 | — | 1 | |
| | 30 | — | 2 | |
| November | 2 | — | 1 | |
| | 10 | — | 1 | |

home, but was ſo ill after ſhe got back into the country, that ſhe was confined for ſeveral days to her bed. The diſeaſe had not then made its appearance in her neighbourhood; but on the fourth day after her return one of her daughters became affected with it, and in the courſe of about three weeks it went through the reſt of her family, which conſiſted of ſix perſons.

In the account given in the London Medical Journal * of the epidemic catarrh of the year 1782, a curious fact was mentioned of its appearance on board two ſhips, from the Weſt Indies, ſoon after their arrival at Graveſend. Mr. Boys, Surgeon at Sandwich, has favoured me with the following account of a fact, of a ſimilar nature, relative to the late epidemic; which is, that "as ſoon as the Roſe "frigate arrived † at Portſmouth from New- "foundland, the dogs ‡ on board were all "ſeized with a cough and catarrh; and ſoon "after-

* Vol. III. page 318.

† The Roſe arrived at Portſmouth on the 4th of November.

‡ Theſe are not the only inſtances of the diſeaſe in dogs that have come to my knowledge. Two dogs belonging to a farmer

" afterwards the whole ſhip's company were " affected in the ſame way." This account Mr. Boys received from his ſon, who is one of the lieutenants of the Roſe.

No period of life ſeemed to be altogether exempt from this diſeaſe; but it ſo happened that I met with very few inſtances of it in children under five years of age, or in perſons of more than ſixty.

Like former epidemics of the ſame kind, it varied conſiderably not only in the ſymptoms that accompanied it in different perſons, but likewiſe in the degree of violence and duration of thoſe ſymptoms, as well as in the order in which they took place. In ſome, who had the diſeaſe very ſlightly, the ſymptoms went off in the courſe of a few hours; but more commonly they laſted ſeveral days, and in many had not entirely ſubſided at the end of a month.

The ſymptoms that occurred the moſt frequently were chillineſs or ſhivering; debility; nauſea and ſometimes vomiting; fever; pains in farmer at Kilburn, and a third, the property of a gentleman at Clapham, died in the month of Auguſt of a diſeaſe that ſeemed clearly to be catarrh; and in all three the throat was much affected.

the back and limbs ; a ſenſation of ſtiffneſs about the neck ; pain in the back part of the head, in one or both ears, in the temples and fore part of the head ; ſneezing ; a diſcharge from the eyes and noſe ; cough ; hoarſeneſs ; ſore throat ; impaired taſte and appetite ; a painful ſenſation about the pit of the ſtomach ; and diarrhœa.

Of theſe different ſymptoms, the only one that could be ſaid to be almoſt conſtantly preſent, at ſome period or other of the diſeaſe, was the pain in the fore part of the head. In ſome this was the firſt ſymptom, coming on inſtantaneouſly with great violence, and accompanied with giddineſs ; but it was not always followed by any conſiderable catarrhal ſymptoms. In ſome inſtances it did not take place till the patient had been ill for ſome days with cough, pain at the ſtomach, and other ſymptoms, and in many caſes was ſo ſlight as hardly to excite attention.

Another ſymptom that occurred very frequently was the pain about the pit of the ſtomach. In ſome the pain extended along the whole courſe of the ſternum, but more commonly it was confined to the part juſt now mentioned, and, when it prevailed in any conſiderable degree, was attended with great anxiety.

This

This painful affection, though it oftentimes ſeemed to be increaſed by the cough, and in ſeveral caſes was accompanied with vomiting and diarrhœa, occurred, however, in many patients who had neither of thoſe ſymptoms.

The cough, though it took place in a great number of inſtances, was ſo far from being a conſtant ſymptom, that, in at leaſt one third of the caſes that came within my obſervation, it made no part of the complaint. Whenever it did occur, it was, at firſt, commonly ſlight; but, by degrees, it became more deeply ſeated, and in many patients was of long continuance, and attended with conſiderable mucous expectoration, which did not eaſily give way to medicines; but I have, as yet, met with no inſtance of its termination in phthiſis.

The diſcharge from the eyes was generally accompanied with redneſs and ſwelling of the eyelids; and in a few inſtances I obſerved a conſiderable degree of ophthalmia.

Sore throat occurred in about one ſixth of the caſes I had occaſion to ſee. In general, where this ſymptom formed a part of the complaint, on inſpecting the fauces, there was obſervable only a redneſs of the mucous membrane. I ſaw no inſtance of ulceration; but in a few caſes the tonſils ſwelled conſiderably, and in two a ſuppuration enſued.

A diarrhœa occurred with much greater frequency than ſore throat, and in a few caſes increaſed to dyſentery. The diarrhœa ſometimes took place early in the diſeaſe, and oftentimes, after having ceaſed for a day or two, returned again; and this, in ſome caſes, repeatedly. In many patients it ſeemed to be the means of diminiſhing the other ſymptoms, and of ſhortening the diſeaſe.

The pulſe, except in the ſeverer forms of the diſeaſe, was ſeldom increaſed to more than a hundred ſtrokes in a minute, but it was commonly diminiſhed in ſtrength; and the patients, in general, were affected with much languor and debility. An increaſe of fever uſually took place towards night, with remiſſion in the morning; and in many caſes there was a great tendency to ſweating. The ſleep was interrupted and unrefreſhing; and in ſeveral inſtances I obſerved a ſlight degree of delirium.

The tongue was ſometimes dry, but more commonly moiſt and covered with a white mucus.

The ſtate of the urine varied according to the degree degree of fever; but I obferved in it nothing that particularly requires to be noticed.

Among the fymptoms that occurred only in a fmall number of the cafes I faw were peripneumony, fwelling of the fubmaxillary glands, fwelling and eryfipelatous inflammation of the face, anafarcous fwelling of the legs, and deafnefs. In one cafe the difeafe, at the end of a week, terminated in an intermittent; in another, an abfcefs took place in one of the ears; and in a third it was attended with a profufe difcharge from the falivary glands.

In feveral cafes, when the difeafe feemed to have almoft entirely fubfided, there fuddenly came on a return of the former complaints, particularly of the head-ach, with increafed violence, and fometimes with the addition of frefh fymptoms. In three inftances fuch a relapfe took place a fecond, and in one even a third time, leaving the unhappy fufferers in a very debilitated ftate.

As in former epidemic catarrhs, fo in this, the number of perfons who died of the difeafe would probably be found to be very fmall compared with the number of thofe who were attacked with it. I heard, indeed, of feveral inftances of its fatal termination; but only two fuch

ſuch occurred among the caſes that came under my immediate inſpection. One of theſe patients was a man of about ſixty years of age, who had for ſeveral years been infirm and aſthmatic; the other was a young man, who had occaſionally been ſubject to hæmoptoe. In both theſe caſes the predominant ſymptom was peripneumony.

In the year 1782 a remarkable increaſe in the burials was found * to take place after the diſeaſe had prevailed about three weeks; and at the period when its effects might be ſuppoſed to be over, the numbers were again reduced to what they were previous to the appearance of the diſeaſe. Nothing like this occurs in the weekly bills of mortality of the preſent year.

In the ſlighter forms of the diſeaſe recourſe was ſeldom had to medical aſſiſtance; and when it prevailed with greater ſeverity the indications of cure, as may be eaſily conceived from the account given of the ſymptoms, were extremely various, though, in general, ſufficiently obvious.

Bleeding did not often appear to be neceſſary. The moſt general indication ſeemed to be to en-

* See Medical Tranſactions, Vol. III. page 78.

courage courage a gentle moiſture on the ſkin by a liberal uſe of tepid diluting liquors, occaſionally aſſiſted by antimonials in ſmall doſes, or the common ſaline draught. Many patients ſeemed to experience good effects from a purgative medicine given early in the diſeaſe; and emetics were commonly of great uſe, both by leſſening the uneaſineſs at the pit of the ſtomach, and by relieving the cheſt in caſes where the patients were troubled with much cough and mucous expectoration.

Opiates were of ſervice in allaying the cough and other effects of irritation, and likewiſe in promoting the determination to the ſkin.

Bliſters became occaſionally neceſſary when the complaint was accompanied with much affection of the throat or cheſt; and likewiſe in a few caſes, where, from the ſtate of the pulſe and other ſymptoms, the patients ſeemed to be in danger of ſinking under the diſeaſe. In theſe laſt I recommended the uſe of wine.

In the courſe of the diſeaſe other remedies became at times neceſſary to regulate the ſtate of the bowels, or to aſſiſt or moderate the expectoration; and when the fever had ſubſided, and the patients recovered ſlowly, the uſual auxiliaries, ſuch as change of air, exerciſe, tonics, nics, &c., were recommended as circumſtances ſeemed to require. In the greater number of caſes, however, even of thoſe in which the diſeaſe had prevailed with a conſiderable degree of ſeverity, I obſerved, that when the ſymptoms had abated, the patients ſpeedily recovered their former ſtate of health.

*December* 3, 1788.

